# Tailored Internet-Delivered Mindfulness-Based Interventions for Patients With Hepatocellular Carcinoma After Transarterial Chemoembolization: Qualitative Study

**DOI:** 10.2196/78337

**Published:** 2026-01-29

**Authors:** Zengxia Liu, Min Li, Yong Jia, Li Chen

**Affiliations:** 1 School of Nursing Anhui Medical University Hefei China; 2 Department of Nursing First Affiliated Hospital of Anhui Medical University Hefei China; 3 School of Nursing Jilin University Changchun, Jilin China; 4 Department of Intervention First Hospital of Jilin University Changchun China

**Keywords:** internet, mindfulness, hepatocellular carcinoma, cancer, qualitative study

## Abstract

**Background:**

Patients with hepatocellular carcinoma (HCC) undergoing transarterial chemoembolization (TACE) experience significant psychological distress, impacting outcomes. While mindfulness-based interventions (MBIs) are beneficial, access is limited. Internet-delivered MBIs (iMBIs) offer an accessible alternative; yet, qualitative understanding of patient experiences with tailored iMBIs for this specific population is lacking.

**Objective:**

This study aimed to explore the facilitators and barriers of patients with HCC post TACE and participated in tailored iMBIs.

**Methods:**

From November 2020 to December 2022, 11 patients with HCC post TACE who had taken part in tailored iMBIs were purposively recruited from a tertiary hospital in Jilin Province. Data were collected through semistructured interviews lasting 30-60 minutes. The interviews were analyzed using conventional content analysis.

**Results:**

Five main categories emerged from the analysis: (1) mindfulness mindset, including acceptance, calmness, and mood improvement; (2) improvement of physical discomfort, such as better sleep, pain relief, reduced gastrointestinal symptoms, and increased activity levels; (3) resistance to mindfulness practice, including perceived lack of effectiveness, unsuitable conditions, equipment limitations, and difficulty concentrating; (4) support and encouragement, involving social support, supervision, and professional guidance; and (5) accessibility and convenience characterized by restoration of life balance and user-friendly features of the practice. Each category encompassed several subcategories reflecting the diverse experiences of participants.

**Conclusions:**

While iMBIs were generally perceived as convenient and accessible, challenges such as equipment limitations were noted. Future implementation should focus on enhancing supportive factors to improve adherence, minimizing barriers, and refining the design and delivery of iMBI programs.

**Trial Registration:**

Chinese Clinical Trial Registry ChiCTR1900027976; https://www.chictr.org.cn/showproj.html?proj=46657

## Introduction

Primary liver cancer is the third leading cause of cancer-related death worldwide and one of the most prevalent malignancies. China accounts for 45.27% of global cases and 47.12% of liver cancer–related deaths [1]. In 2022, there were approximately 368,000 new cases and 317,000 deaths from liver cancer in China, ranking fourth in cancer incidence and second in cancer-related mortality [2]. Hepatocellular carcinoma (HCC) is the most common histological subtype of primary liver cancer, comprising 75%-85% of all cases [3]. Transarterial chemoembolization (TACE) is a widely accepted local treatment for HCC and is the preferred option for patients with intermediate-stage HCC who are not suitable candidates for surgery [4,5]. However, patients often require multiple TACE sessions to manage disease progression. Despite treatment, they continue to face high risks of recurrence, metastasis, and various complications [6]. Moreover, the effectiveness of TACE diminishes with repeated use, and frequent treatments can result in progressive liver damage [7].

Due to the high mortality rate of HCC, research showed that the 5-year survival rate of patients with HCC after diagnosis was only 19.5% [8].Therefore, most patients with HCC face a serious threat of death, and their psychological distress is generally higher than that of other patients with cancer. During TACE treatment, some adverse reactions may occur, such as nausea, vomiting, fever, and pain. TACE patients have to face not only the physical pain brought by the disease and treatment but also the fear of disease recurrence, the high medical costs of repeated TACE treatments, and the pressure of being unable to return to society. This causes them to bear tremendous mental stress, resulting in widespread and severe psychological distress among patients with HCC post TACE [9,10]. This psychological burden can weaken patients’ immune response, reduce treatment compliance, and adversely affect treatment outcomes, ultimately increasing the likelihood of tumor recurrence, deterioration, and metastasis [11].

Psychological distress in patients with cancer often arises not from a single external stressor that can be resolved or avoided but from the persistent fear of recurrence. Coping effectively, therefore, requires adapting to this ongoing internal experience. Mindfulness-based interventions (MBIs), which are designed to help individuals manage distressing thoughts and emotions, are particularly well suited for this purpose. The effectiveness of MBIs in alleviating psychological distress among patients with cancer has been well documented [12,13]. However, patients with HCC post TACE face unique physiological and psychological challenges. The uncertainty surrounding treatment outcomes, fear of recurrence, financial burden of repeated treatments, and treatment-related side effects collectively inflict significant physical and emotional strain. These factors highlight the urgent need to develop tailored MBIs specifically for this population—interventions that take into account not only the psychological profile and coping mechanisms of patients with HCC but also their physical health status. Despite the proven benefits of MBIs, several barriers limit access, including a shortage of trained therapists, high costs, and practical constraints, such as limited mobility and time availability among patients with cancer [14]. Addressing these challenges is essential to ensure that MBIs are accessible and effective for those who need them most.

Internet-delivered mindfulness-based interventions (iMBIs) offer a promising solution to many of the barriers associated with accessing psychosocial care in cancer treatment settings. Compared with traditional face-to-face interventions, iMBIs have several advantages. They can be accessed online at any time, allowing participants to engage with the program at their own convenience. This flexibility is particularly beneficial for patients with HCC and helps overcome practical challenges such as geographical distance, transportation difficulties, cancer-related fatigue, and mobility issues. This flexibility is particularly beneficial for patients with HCC post TACE, as most of them have a heavy burden of physical symptoms. This method helps overcome practical challenges such as geographic distance, transportation difficulties, cancer-related fatigue, and limited mobility. While iMBIs are associated with a higher rate of participant dropout, studies have nonetheless demonstrated their effectiveness in reducing psychological distress among patients with cancer [15-17].

Most studies investigating iMBIs in patients with cancer rely on standardized questionnaires to assess outcomes [18-20]. However, such quantitative approaches fail to capture the personal experiences and perceptions of participants. These subjective experiences are difficult to evaluate through usage data or survey metrics alone. Therefore, the primary aim of this qualitative study was to explore participants’ experiences and perceived effects of tailored iMBIs. This approach allows for a deeper understanding of their thoughts and feelings, a comprehensive examination of both the benefits and the challenges of the intervention, and an analysis of factors that influence participant adherence and engagement.

## Methods

### Research Design

To gain a deep understanding of participants’ experiences with iMBIs, as well as the interventions’ acceptability and applicability, a qualitative descriptive design using semistructured personal interviews was used. The study followed the COREQ (Consolidated Criteria for Reporting Qualitative Research) [21].

### Participants

From November 2020 to December 2022, the study was conducted at the First Hospital of Jilin University. Potential participants were identified by evaluating and screening patient medical records to determine eligibility based on the inclusion criteria. The inclusion criteria for participants were as follows: (1) aged 18 years or older; (2) diagnosed with HCC based on the diagnostic criteria of the European Association for the Study of the Liver [22]; (3) currently received TACE; (4) able to operate a smartphone and use WeChat frequently (at least 5 times per week); (5) able to read, write, and speak Chinese; and (6) provided informed consent and voluntarily agreed to participate in the study. The exclusion criteria for participants were as follows: (1) diagnosed with other types of tumors or mental stress disorders, (2) taking psychotropic medications during the study period, (3) received mindfulness intervention, and (4) experiencing poor health that interferes with normal communication. Those who met the criteria were invited to participate in a mindfulness intervention. Interviewees were then selected from among the participants who completed the iMBIs intervention.

In accordance with the principle of maximum variation sampling, participants were chosen to reflect a wide range of differences in age, gender, education level, and frequency of TACE treatments. The sample size was determined according to the concept of data saturation, meaning that collection ceased once no new codes emerged and the data appeared to have stabilized. After 11 patients were interviewed and the data were analyzed, the research team agreed that data saturation had been reached, and data collection was stopped.

### Interventions

#### Overview

A cross-sectional survey of patients post TACE revealed that trait mindfulness, perceived stress, and experiential avoidance are potential psychological mechanisms influencing anxiety and depression in patients with HCC [10]. Based on this finding, the research team developed a tailored MBI for patients with HCC post TACE. The intervention was informed by the core principles of Mindfulness-Based Stress Reduction (MBSR) [23], Acceptance and Commitment Therapy [24,25], and Mindfulness-Based Cancer Recovery, as well as insights from a systematic review and an assessment of the characteristics and internet usage habits of this patient population. An initial draft of the tailored MBI was created and then refined through expert consultation and revisions.

**Figure 1:**
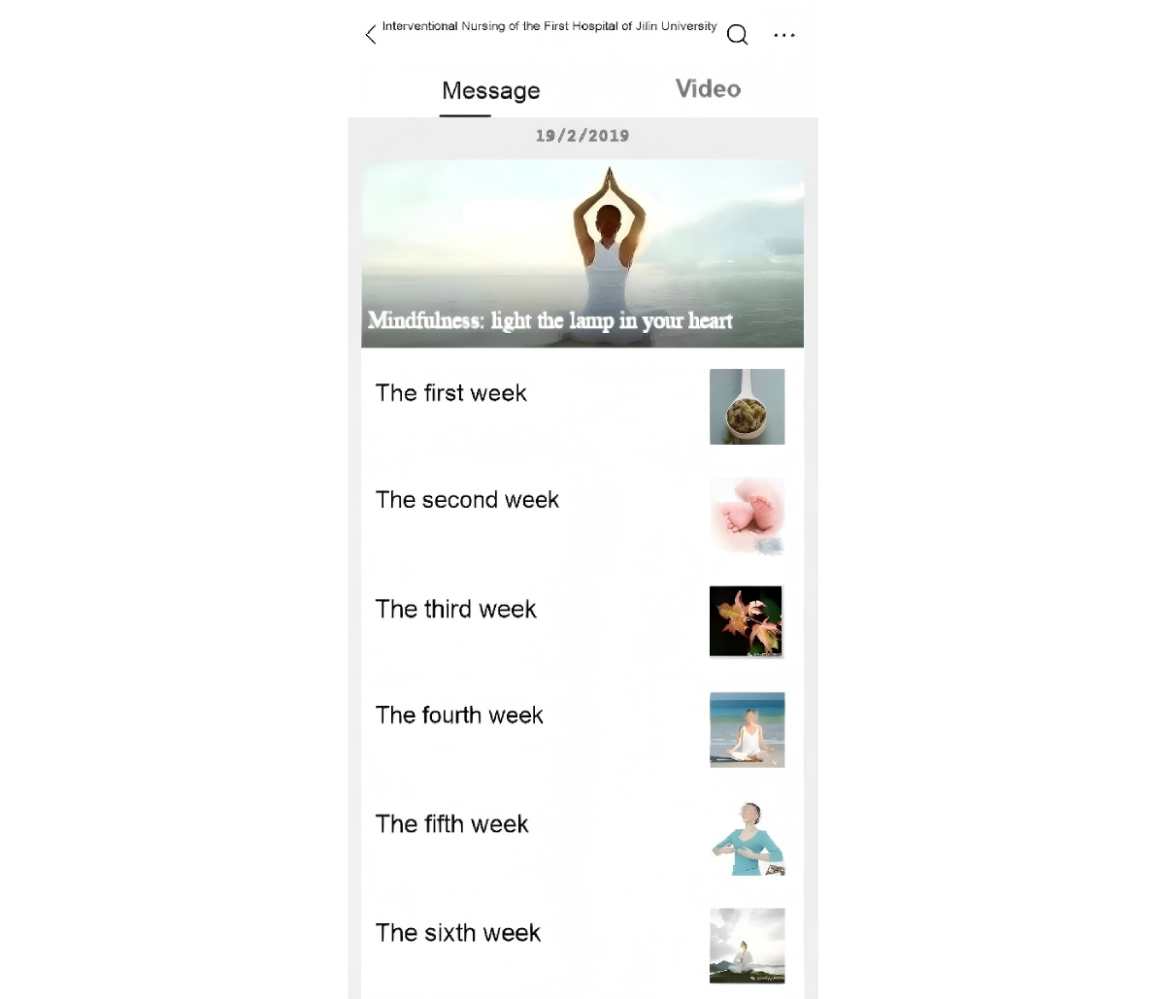
The interface of the WeChat official account for the intervention content.

A self-guided intervention was delivered through WeChat groups and an official WeChat account, which was used to distribute weekly mindfulness content. This content was presented in various formats, including text, audio, video, and images, via the official WeChat official account. The interface of the WeChat official account for the intervention content is shown in Figure 1. The intervention lasted for 6 weeks and included an initial in-hospital face-to-face session during the first week, followed by web-based sessions from the second to the sixth week.

#### In-Hospital Face-to-Face Intervention (Week 1)

The face-to-face sessions were conducted in the interventional department classroom, with 5-8 participants per group. Each session lasted 1-1.5 hours. During this session, the research team introduced the study’s purpose and content using presentation slides, provided an overview of mindfulness and its benefits, and helped participants understand the attitudes and principles underlying mindfulness practice. The session also familiarized participants with the structure and content of the upcoming web-based intervention, ensuring that they were prepared for the self-guided practice in the following weeks.

#### Out-of-Hospital Web-Based Intervention (Weeks 2-6)

For the remainder of the intervention, content was delivered weekly through WeChat in the form of audio, video, or image-based materials. Participants were encouraged to engage in daily mindfulness practice for 10-20 minutes, 5 days per week. The research team provided regular reminders and encouragement via WeChat and phone calls to ensure adherence. Participants were also encouraged to “check in” daily in the WeChat group by sharing their experiences and reflections on mindfulness practice. In addition, the research team shared educational materials through the WeChat group, covering topics such as mindfulness, HCC, and TACE treatment. They also responded to participants’ questions regarding the disease, mindfulness practices, and other related concerns. The specific intervention content is detailed in Table 1.

Before interventions, 5 patients were invited to conduct a pre-experiment. After the first 2 weeks of intervention, adjustments were made according to the participants’ feedback. Participants reported that the text on the official account interface was too small, which was not conducive to reading. Therefore, the researchers reorganized the layout to make the interface clearer and more esthetically pleasing. Participants also mentioned that there was too much text content, which was not conducive to reading and practice. As a result, the researchers converted some of the text content into audio to facilitate participants’ learning and practice. Participants suggested that more pictures and videos could be added to make the content more vivid. The researchers added some pictures and video content to make the content more vivid and practical.

**Table 1:** The tailored internet-delivered mindfulness-based interventions for patients with HCC^a^ post TACE^b^.

Week	Theme of intervention	Summary of contents	Homework	Intervention mode
Week 1	Mindfulness and HCC and self-acceptance	1. Knowledge introduction: Introducing content related to mindfulness, HCC and TACE treatment, and the relationship between mindfulness and cancer. 2. Mindful breathing: Guide participants to practice mindful breathing, exhaling and inhaling easily, experiencing every detail of breathing, including the process, changes and pauses of breathing, accepting their breathing state, and being aware of the changes in their thoughts and attention. 3. “The Missing Piece”: Watch the video “The Missing Corner” to prompt participants to think about “unchangeable imperfections,” learn to accept negative events or emotions in life, and encourage participants to share their feelings after watching.	Mindful breathing	Face-to-face intervention in the hospital, 5-8 participants per group, 1-1.5 hours per session, introducing the purpose and content of the study, and guiding the completion of the first session.
Week 2	Cancer and stress and cognitive dissociation	1. Knowledge introduction: Describe knowledge related to cancer and stress, and typical stress reactions to cancer stress events. 2. Body scan: Introduce the connotation and requirements of body scan. Guide participants to feel and experience the sensation of each part of the body in order from feet to head. Pay attention to the feelings of each part, whether it is comfortable or not. Do not judge. Emphasize full commitment to the body-scanning practice. 3. “I have such an idea now”: Guide participants to practice “I have such an idea now.” Ask the participants to state a thought that makes them depressed, such as “Why did I get liver cancer?” Repeat this several times. After 3 minutes, add “I have an idea. Why did I get liver cancer?” before this thought. Repeat this several times. After 3 minutes, add a sentence before this idea, “I noticed that I have an idea. Why did I get liver cancer?” The main purpose of this exercise is to distinguish the real self from the imagined self, achieve cognitive dissociation, and prevent these thoughts from affecting an individual’s life.	Body scan	Out-of-hospital online intervention, 20 minutes per day, at least 5 days per week, learn the intervention content via official account and WeChat, and 10 minutes of homework practice.
Week 3	Identifying avoidance reactions and self-awareness	1. Meditation practice: Guide participants to practice mindful meditation. Be aware of the thoughts and emotions that enter your mind. Pay attention to these thoughts and emotions but do not focus too much on them. Try to let go and shift your attention to your breathing. Then slowly shift your attention to your body, voice, thoughts, and emotions, and return to your breathing. In this repetitive process, gradually become aware of and feel your thoughts and emotions, and gradually and spontaneously coexist with them. 2. “Chessboard metaphor”: Guide participants to imagine a “chess game.” No longer caring about winning or losing, just providing a venue for the game of chess, as an observer, to observe the competition. Through the “chessboard metaphor,” participants are guided to stop getting entangled in their inner struggles, become observers of their own psychology, and achieve self-awareness.	Meditation practice	Out-of-hospital online intervention, 20 minutes per day, at least 5 days per week, learn the intervention content via official account and WeChat, and 10 minutes of homework practice.
Week 4	Practice mindfulness in life, focusing on the present	1. Mindfulness walking: Guide patients to practice mindfulness walking and feel the current movements while practicing. From the lifting of the foot to the forward movement and the landing process, the sensation of each part of the foot, when practicing, feel the current movement and the current feeling, and focus your attention on the action of mindful walking. 2. Mindfulness eating: Guide patients to practice mindfulness eating. Slow down your eating speed, focus on the movements and sensations of your tongue and mouth while eating, experience the changes and flavors of food in your mouth, and pay attention to observing your inner feelings and emotions during the practice.	Mindful walking	Out-of-hospital online intervention, 20 minutes per day, at least 5 days per week, learn the intervention content via official account and WeChat, and 10 minutes of homework practice.
Week 5	Nonselective awareness, clear value	1. Awareness practice: Explain the key points of awareness practice to patients and be aware of everything around them. Perceive the things around you and the sensations of your body with an accepting attitude, including pain, fatigue, and so on, and embrace everything. In practice, no matter what you perceive, trust and accept yourself. 2. “Compass” metaphor: Guide participants to think about the guiding role of the “compass” in the journey. By using this metaphor for goals and values, it guides participants to think about the direction and goals they want to move forward in, and based on their own values, they choose to set a small goal and take action.	Awareness practice	Out-of-hospital online intervention, 20 minutes per day, at least 5 days per week, learn the intervention content via official account and WeChat, and 10 minutes of homework practice.
Week 6	Grateful life, committed to action	1. Love meditation, good wishes: Cultivate the mindfulness practice of kindness and bestow your blessings upon others during the practice. You can choose different blessings in sequence to send to different people, including yourself, your family, friends, or even someone you dislike. Fill the participants’ hearts with tolerance and love. 2. “Passengers on the Bus”: Through the short film “Passenger Practice on the Bus,” participants are guided to compare their own lives to buses and think about how to keep moving forward on the path of happiness when encountering different passengers, such as “stress,” “anxiety,” “happiness,” and so forth, thereby contemplating the significance of committing to action.	Love meditation	Out-of-hospital online intervention, 20 minutes per day, at least 5 days per week, learn the intervention content via official account and WeChat, and 10 minutes of homework practice.

^a^HCC: hepatocellular carcinoma.

^b^TACE: transarterial chemoembolization.

### Data Collection

Based on the disease characteristics and treatment experiences of patients with HCC following TACE, a preliminary interview guide was developed through a literature review and discussions among the research team. Prior to the formal interviews, 2 participants were selected for pilot interviews. The final interview guide was revised based on the issues identified during these pilot sessions and is shown in Table 2. Demographic information was collected before the interviews.

Two researchers (ZL and YJ), graduate students pursuing a Doctor of Nursing degree, approached potential participants during conducting the research. Once the patients had completed their consultations and treatments, we invited them to participate in this study. If they agreed to participate, we clearly explained the study’s purpose and procedures to them. The researchers coordinated with participants in advance to schedule interviews and conducted them in a private room at the hospital to ensure confidentiality. At the beginning of each session, the researchers introduced themselves and explained the purpose, methods, and content of the interview. Participants were informed that the interview would be audio-recorded to ensure the completeness and accuracy of the data. Informed consent was obtained prior to starting the interview. During the interviews, the researchers maintained a neutral and nondirective attitude, using appropriate and nonleading language to avoid influencing participants’ responses. Data collection and analysis were carried out concurrently to support iterative refinement of the findings.

During the interview, we used the interview tools proposed by Robinson [26], which include descriptive, individualized memory, exploratory, and clarifying inquiries. The individualized memory inquiry was used to guide the participants in recalling specific periods to obtain detailed information, such as the duration of their participation in iMBIs. The descriptive inquiry was used to explore the participants’ feelings of participation. The interpretive inquiry was used to reveal the participants’ views on iMBIs. The clarifying inquiry was used to clarify keywords and the implicit meanings of expressions. Based on the participants’ responses, we modified or omitted the interview questions, or introduced new questions to explore new emerging topics, such as the influence of family on the participants’ participation in iMBIs.

**Table 2:** Interview outline.

Number	Question
1	Why did you participate in this network-based mindfulness training?
2	What are your experiences while participating in this training?
3	What effects does network-based mindfulness training have on your body?
4	How does the network-based mindfulness training affect you psychology?

### Ethical Considerations

The study was executed in accordance with the principles of the Declaration of Helsinki. The research protocol was approved by the ethics committee of the School of Nursing at Jilin University (approval no. 2019112001) and was registered with the Chinese Clinical Trial Registration Center (ChiCTR1900027976) on December 7, 2019. Prior to the interviews, participants were informed about the purpose and content of the study. They were assured that all data would be used solely for research and publication purposes, and that their personal information would remain confidential. Participants were also informed of their right to withdraw from the study at any time without any consequences. Written informed consent was obtained from all participants before the study commenced.

### Data Analysis

One researcher (LZ) participating in the interview listened to the recordings repeatedly within 24 hours after each interview and transcribed them into text. Another researcher (LM) participating in the interview checked the transcribed text and imported it into NVivo 11 software [27] for analysis. Conventional content analysis [28,29] was used to analyze the data. Three researchers (LZ, JY, and CL) independently analyzed and transcribed the data.

### Methodological Rigor and Trustworthiness of Data

Members of the research team include liver cancer clinical experts, nursing specialists, and psychologists. Interviews were conducted by 2 researchers (LZ and LM) to avoid information omission. To establish credibility, we used both analyst triangulation and data triangulation. Analyst triangulation was used to ensure intersubjective stability of the results and involved the independent analysis of the data by 3 researchers (LZ, JY, and CL), followed by a comparison of the analysis. The data were collated and analyzed by experienced researchers in qualitative research, the speech information of the interviewees was comprehensively retained, and complete and detailed textual data were established. The 2 researchers (LZ and JY) organized, analyzed, and transcribed the data independently. If there were differences, they were resolved through discussion or consultation with the third researcher (CL) until the results were reached.

## Results

### Characteristics of Participants

In this study, interviews were conducted with 9 participants, during which no new information emerged. To confirm data saturation, interviews with 2 additional participants were carried out, and again, no new themes were identified. Thus, a total of 11 participants were included in the final analysis. Their demographic and clinical characteristics are shown in Table 3.

**Table 3:** Characteristics of semidepth interview participants (N=11).

Number	Age (years)	Sex	Education level	Number of TACEs^a^
1	38	Male	Senior high school	1
2	50	Male	College school	8
3	53	Female	Senior high school	1
4	42	Female	College school	4
5	61	Male	Primary school	5
6	57	Female	Senior high school	1
7	49	Female	College school	7
8	62	Male	Senior high school	3
9	63	Female	Senior high school	3
10	48	Male	College school	7
11	40	Male	Primary school	4

^a^TACE: transarterial chemoembolization.

### Categories

#### Overview

Through repeated analysis and organization of the interview data, 5 main categories reflecting the participants’ experiences were identified: mindfulness mindset, improvement of physical discomfort, resistance to mindfulness practice, support and encouragement, and accessibility and convenience. Each main category is further divided into several subcategories, as shown in Textbox 1.

**Relevant categories corresponding to experiences of participants.**

1. **Mindful mindset**
1.1 Acceptance
1.2 Calm
1.3 Mood improvement
    1.3.1 Relaxation
    1.3.2 Gratitude
2. **Improvement of physical discomfort**
2.1 Improve sleep
2.2 Pain relief
2.3 Reduce gastrointestinal symptoms
2.4 Increase activities
3. **Mindfulness practice resistance**
3.1 Lack of effect
  3.2 Condition
    3.2.1 Fatigue
    3.2.2 Pain
3.3 Device usage restrictions
3.4 Difficulty focusing
3.5 Lack of motivation to practice
4. **Support and encourage**
4.1 Social support
4.2 Supervision and guidance
5. **Accessibility and convenience**
5.1 Restore a sense of balance in your life
5.2 Practice features

#### Main Category 1: Mindfulness Mindset

The mindfulness mindset refers to the ability of patients with HCC following TACE to face their condition with a nonjudgmental attitude, while maintaining a curious, open, and accepting approach toward their present thoughts and emotions.

##### General Category 1.1: Acceptance

Six participants reported that practicing mindfulness helped them adopt a more accepting attitude toward their illness and the challenges they faced in life.

My mother and I both have hepatitis, and my mother died of HCC, so I’ve always been worried about my health. I get scared every time I go for a check-up. But after doing the mindfulness exercises, my mindset changed. I gradually realized that I shouldn’t overthink things every day. Since I have this disease, I just need to follow the doctor’s advice and receive treatment. Many treatments are effective, but overthinking isn’t good for my health.

Interview ig4

##### General Category 1.2: Calm

Seven participants reported that after practicing mindfulness, they experienced a greater sense of inner calm and became more attuned to the beauty in their surroundings.

My mindset has improved a lot. I can now perceive things around me more calmly and appreciate the little moments in life. I’m more willing to take walks, enjoy the scenery, and notice the beauty in simple things, like a tree or a flower by the roadside.

Interview ig9

##### General Category 1.3: Mood Improvement

###### Subcategory 1.3.1: Relaxation

Five participants shared that mindfulness practice helped them better regulate their emotions, reduce anxiety, and achieve a more relaxed state of mind.

Every time I went for a check-up, I would get really nervous if there was any change in my test results. Even small changes would worry me for a long time. Even when the doctor reassured me that everything was fine, I would still look up all kinds of information online. Now, when I notice myself feeling anxious, I try to adjust my emotions, relax, and avoid overthinking things that haven't even happened.

Interview ig4

###### Subcategory 1.3.2: Gratitude

Two participants expressed gratitude. After practicing mindfulness, they were able to approach people and situations around them with a more thankful attitude.

I’ve always had a bad temper and often got angry. My family would give in to me because of my poor health, which made me even angrier. After listening to the exercises, my mindset gradually improved. Now, when I encounter unpleasant things, I can stay calm. I also recognize how much my family has sacrificed for me, and my attitude has changed a lot. The atmosphere at home is much better.

Interview ig3

#### Main Category 2: Improvement of Physical Discomfort

Improvement of physical discomfort refers to the reduction of physical symptoms and alleviation of discomfort experienced by patients with HCC after TACE.

##### General Category 2.1: Improve Sleep

Ten participants reported significant improvements in sleep quality after mindfulness practice, with less presleep rumination and easier time falling asleep.

I’ve always had poor sleep, often lying in bed for a long time without falling asleep. The worse my sleep, the more I get lost in my thoughts. But since I started these exercises, I fall asleep shortly after listening. It helps stop my random thoughts.

Interview ig3

##### General Category 2.2: Pain Relief

Three participants said that mindfulness helped relieve pain and reduce physical discomfort.

I used to feel discomfort all over my body and pain everywhere. During the exercises, I take deep breaths, relax, follow the teacher, and slowly let go of tension. These uncomfortable feelings fade away, and I feel much better.

Interview ig6

##### General Category 2.3: Alleviation of Gastrointestinal Symptoms

Two participants reported that mindfulness practice helped ease gastrointestinal symptoms, improved their appetite, and reduced bloating.

I often had no appetite, felt uncomfortable after eating, and experienced bloating. Now I do these exercises daily, and these discomforts have improved. Sitting and relaxing each day reduces my irritability and unease.

Interview ig1

##### General Category 2.4: Increase Activities

Two participants noted that mindfulness helped reduce fatigue and encouraged them to be more active.

My whole body felt weak. I would just sit or lie down, unwilling to do anything. Sometimes I felt exhausted for long periods. After starting these exercises and practicing regularly, I feel much better. I’m more willing to move and have things to do.

Interview ig4

#### Main Category 3: Resistance to Mindfulness Practice

Resistance refers to factors that hinder patients’ ability to consistently participate in mindfulness practice during the intervention.

##### General Category 3.1: Lack of Effectiveness

Two participants felt that the intervention had little effect on their psychological or physical state.

With this disease, it feels like a death sentence. I wanted to give up, but my family insisted I continue treatment. It feels like all efforts are just a loss of life and money, and this practice doesn’t help.

Interview ig1

##### General Category 3.2: Conditions

###### Subcategory 3.2.1: Fatigue

Three participants reported that fatigue and physical discomfort made it difficult to practice mindfulness regularly.

I have poor appetite and low energy every day. I feel uncomfortable all over, the treatment isn’t working, and I just want to lie down all day without doing anything. It’s hard to practice.

Interview ig4

###### Subcategory 3.2.2: Pain

Two participants said that pain interfered with their ability to engage in mindfulness.

This pain is torturous. I’m not in the mood for anything, and it hasn’t gotten better. When will I get better? This disease is unbearable.

Interview ig6

##### General Category 3.3: Device Usage Restrictions

Two participants said that distractions from their phones prevented them from completing mindfulness exercises consistently.

My phone keeps buzzing with messages, interrupting my practice. It’s hard to stay quiet and focus for even a short time. There are just too many distractions.

Interview ig10

##### General Category 3.4: Difficulty in Focusing

Two participants found it hard to concentrate during mindfulness practice, leading to distraction and difficulty persisting.

When practicing at home, I often got lost in my thoughts. For example, when asked to focus on my abdomen, I’d start worrying about my illness and other messy things. It was hard to fully follow and stick with it.

Interview ig3

##### General Category 3.5: Lack of Motivation to Practice

Two participants reported difficulty maintaining regular practice due to low motivation.

I feel irritable and tired every day. I kept up with the exercises for a few days, but then felt it didn’t work. Many times I was just too lazy to do the exercises.

Interview ig1

#### Main Category 4: Support and Encourage

Support and encouragement refer to the social and professional support received by patients during the online mindfulness intervention.

##### General Category 4.1: Social Support

Two participants shared that family involvement and communication through WeChat groups helped them persevere.

My wife started practicing with me in the hospital and continued after we got home. We share our experiences and feelings. Sometimes I don’t want to practice, but she encourages me. Practicing together helped me stick with it.

Interview ig5

##### General Category 4.2: Supervision and Guidance

Three participants found that daily check-ins and reminders in the WeChat group, along with exchanges with other patients and medical staff, motivated their persistence.

Everyone checked in daily and shared their feelings in the WeChat group. Seeing fellow patients from my ward stick with it and ask questions encouraged me. After more communication, I kept going.

Interview ig2

#### Main Category 5: Accessibility and Convenience

Accessibility and convenience refer to patients’ perceptions of how easy and convenient the online mindfulness intervention was to use.

##### General Category 5.1: Restore a Sense of Balance in Your Life

Three participants said that practicing mindfulness at the same time and place daily helped regulate their lives and restore balance and encouraged ongoing practice.

Since my diagnosis, my family has taken special care of me, not letting me do housework. I just lay around and felt useless. After practicing daily, I feel like I’ve accomplished something and have more energy.

Interview ig4

##### General Category 5.2: Practice Features

Three participants appreciated the convenience of the online format, finding it time-saving, flexible, and easy to fit into their daily routines.

I live in a rural area, and it’s always hard to come to the hospital, especially without family to accompany me. Now I can practice anytime online after finding the audio. The WeChat method is convenient. When I feel down or have free time, I just practice—it doesn’t have to be at a fixed time, so it’s more flexible.

Interview ig11

## Discussion

### Principal Findings

This study found that participants experienced both physical and psychological benefits from the intervention. The physical improvements were primarily related to the alleviation of physical symptoms, consistent with findings from Nissen et al [20], who reported that iMBIs can enhance sleep and increase activity levels in patients with cancer. From a psychological perspective, studies by Eyles et al [30] and Weitz et al [31] demonstrated that an 8-week MBSR program can improve the mental health of patients with breast cancer, helping them live more fully in the present. Living in the present is associated with letting go of anxiety and rediscovering happiness [24]. Mindfulness interventions may also lead to changes in neurophysiological activity, brain structure, and function, which contribute to the positive psychological experiences reported by patients with cancer [32]. The intervention program of this study was based on the relevant contents of MBSR, Acceptance and Commitment Therapy, and Mindfulness-Based Cancer Recovery. Participants can have a better understanding of their own diseases and reduce the worries caused by the lack of disease knowledge. The intervention program incorporated elements of acceptance and gratitude. After the intervention, participants reduced their evasive attitude toward some negative emotions and events, faced negative thoughts and feelings with an accepting attitude, improved psychological flexibility, and thereby reduced anxiety and depression. Participants can better maintain a nonjudgmental mindset to continuously perceive and focus on their current experiences, avoiding their consciousness from diverging and wandering in the virtual world of thought, achieving the goal of stopping distractions, concentrating on real things, and thus attaining mental liberation, thereby enhancing trait mindfulness. These outcomes align with the mindfulness-to-meaning theory proposed by Garland et al [33], which suggests that MBIs enhance trait mindfulness and reduce psychological distress.

Social support and guidance were significant facilitators of mindfulness practice. The WeChat group used in this study provided a platform for participants to share experiences and feelings about mindfulness practice and their illness. The involvement of medical staff enabled timely professional guidance, enhancing participants’ confidence and adherence. The group check-ins and communication encouraged mutual supervision, motivating participants to practice consistently. During the initial in-hospital phase, mindfulness was introduced to both patients and their families, encouraging joint participation, which helped mitigate the limitations of a solely online intervention. Research by Zulman et al [34] supports this approach, showing that engaging both patients with cancer and their caregivers can foster better communication and mutual support.

However, participants also encountered several obstacles, including perceived ineffectiveness, illness-related fatigue, equipment limitations, difficulty concentrating, lack of motivation, and challenges completing practices on time. These barriers may stem from the advanced disease stage and poor health status of patients with HCC [35], which can hinder the ability to engage in even simple exercises. Future intervention designs should consider the patient’s physical condition and create more tailored programs. A lack of professional mindfulness guidance also contributed to reduced compliance. Although this study used health care staff as facilitators, guidance provided through WeChat lacked real-time responsiveness and personalization, which likely weakened adherence. Previous research has shown that iMBIs with interactive guidance have significantly stronger effects on mindfulness and stress reduction than unguided interventions [36,37].

Participant inclusion criteria also influenced outcomes. High or low baseline levels of negative emotions can reduce intervention effectiveness. For example, Compen et al [15] used a Hospital Anxiety and Depression Scale cutoff of ≥11 and reported significantly better results. If patients are already in a stable psychological state, mindfulness interventions may unintentionally reinforce their identity as “patients,” potentially reducing effectiveness. Therefore, selecting participants with appropriate levels of psychological distress is crucial to avoid ceiling or floor effects and to enhance the efficacy of the intervention.

### Implications for Practice and Research

The effectiveness of iMBIs is closely tied to the platform used for delivery. Future iMBI platforms should be designed to meet users’ individual and shared needs, enhancing usability and comfort. Comprehensive mobile health apps tailored for patients post TACE could be further developed. For instance, Subnis et al [38] created a mindfulness app that offers guided meditations, audio lectures, timers, logs, and stress assessments using facial biosignals. Such tools can reduce medical costs and address patients’ psychological needs, providing a sustainable platform for continuous support.

### Strengths and Limitations

This study tailored the iMBI to the physiological and psychological characteristics of participants and delivered it via WeChat, in line with their internet usage habits. Participants appreciated the convenience and flexibility of being able to practice at home, which reduced travel-related burdens. The study also emphasized family involvement, which is particularly relevant in the Chinese cultural context where family support plays a central role.

However, limitations remain. Compared with face-to-face mindfulness interventions, iMBIs lack real-time group interaction and peer support. Although WeChat enabled communication, it was still somewhat limited. Using health care providers instead of trained mindfulness instructors meant that the guidance lacked expertise and real-time feedback. Moreover, the WeChat platform itself had functional limitations, lacking features such as real-time monitoring, personalized feedback, and automatic recording of practice sessions. Another limitation is that interviews were conducted postintervention, relying on participants’ recollections, which may introduce recall bias. This method also did not allow for tracking participants’ evolving experiences over the course of the intervention.

### Conclusions

This study used semistructured interviews to explore participants’ experiences with iMBIs. Participants reported a variety of benefits, particularly psychological ones. Nevertheless, they also conveyed negative experiences, such as ineffectiveness, emotional resistance, difficulty concentrating, and low motivation. Factors such as social support, supervision, and restored life balance promoted adherence, while device-related distractions and the emotional reminder of illness hindered it. Although iMBIs offer convenience and accessibility, there are still important issues to address. Future efforts should explore participants’ individualized needs more deeply and develop comprehensive, user-friendly apps to enhance both the comfort and the effectiveness of mindfulness interventions.

## Appendix

Multimedia Appendix 1 COREQ checklist.

## Abbreviations

COREQ: Consolidated Criteria for Reporting Qualitative Research
HCC: hepatocellular carcinoma
iMBIs: internet-delivered mindfulness-based interventions
MBI: mindfulness-based intervention
MBSR: Mindfulness-Based Stress Reduction
TACE: transarterial chemoembolization

## Data Availability

The data are not publicly available because they contain information that could compromise the privacy of study participants but are available from the corresponding author on reasonable request.
