# Supplementary material for: Tailored Internet-Delivered Mindfulness-Based Interventions for Patients With Hepatocellular Carcinoma After Transarterial Chemoembolization: Qualitative Study
Source: J Med Internet Res. 2026 Jan 29;28:e78337. doi: 10.2196/78337 (PMC12902756; doi:10.2196/78337)
Supplement: Multimedia Appendix 1 [file jmir_v28i1e78337_app1.pdf]

# COREQ (COnsolidated criteria for REporting Qualitative research) Checklist

| Topic | Item No. | Guide Questions/Description | Reported on Page No. |
|---|---|---|---|
| **Domain 1: Research team and reflexivity** | | | |
| *Personal characteristics* | | | |
| Interviewer/facilitator | 1 | Which author/s conducted the interview or focus group? | Page 10 |
| Credentials | 2 | What were the researcher's credentials? E.g. PhD, MD | Page 10 |
| Occupation | 3 | What was their occupation at the time of the study? | Page 10 |
| Gender | 4 | Was the researcher male or female? | Page 10 |
| Experience and training | 5 | What experience or training did the researcher have? | Page 10 |
| *Relationship with participants* | | | |
| Relationship established | 6 | Was a relationship established prior to study commencement? | Page 10-11 |
| Participant knowledge of the interviewer | 7 | What did the participants know about the researcher? e.g. personal goals, reasons for doing the research | Page 10-11 |
| Interviewer characteristics | 8 | What characteristics were reported about the inter viewer/facilitator? e.g. Bias, assumptions, reasons and interests in the research topic | page 10-12 |
| **Domain 2: Study design** | | | |
| *Theoretical framework* | | | |
| Methodological orientationand Theory | 9 | What methodological orientation was stated to underpin the study? e.g.grounded theory, discourse analysis, ethnography, phenomenology, content analysis | Page 12 |
| *Participant selection* | | | |
| Sampling | 10 | How were participants selected? e.g. purposive, convenience, consecutive, snowball | Page12 |
| Method of approach | 11 | How were participants approached? e.g. face-to-face, telephone, mail, email | Page 11 |
| Sample size | 12 | How many participants were in the study? | Page 12 |
| Non-participation | 13 | How many people refused to participate or dropped out? Reasons? | Page 12 |
| Setting | | | |
| Setting of data collection | 14 | Where was the data collected? e.g. home, clinic, workplace | Page 11 |
| Presence of non-participants | 15 | Was anyone else present besides the participants and researchers? | Page11 |

| | | | |
|---|---|---|---|
| Description of sample | 16 | What are the important characteristics of the sample? e.g. demographic data, date | Page13 |
| Data collection | | | |
| Interview guide | 17 | Were questions, prompts, guides provided by the authors? Was it pilot tested? | Page 10-11 |
| Repeat interviews | 18 | Were repeat inter views carried out? If yes, how many? | Page 10-11 |
| Audio/visual recording | 19 | Did the research use audio or visual recording to collect the data? | Page 10-11 |
| Field notes | 20 | Were field notes made during and/or after the inter view or focus group? | Page10-11 |
| Duration | 21 | What was the duration of the inter views or focus group? | Page 10-11 |
| Data saturation | 22 | Was data saturation discussed? | Page 10-11 |
| Transcripts returned | 23 | Were transcripts returned to participants for comment and/or correction? | Page 10-11 |
| **Domain 3: analysis and findings** | | | |
| *Data analysis* | | | |
| Number of data coders | 24 | How many data coders coded the data? | Page 12 |
| Description of the coding tree | 25 | Did authors provide a description of the coding tree? | Page 12 |
| Derivation of themes | 26 | Were themes identified in advance or derived from the data? | Page 12 |
| Software | 27 | What software, if applicable, was used to manage the data? | Page 12 |
| Participant checking | 28 | Did participants provide feedback on the findings? | Page 11 |
| *Reporting* | | | |
| Quotations presented | 29 | Were participant quotations presented to illustrate the themes/findings? Was each quotation identified? e.g. participant number | Page 14- 19 |
| Data and findings consistent | 30 | Was there consistency between the data presented and the findings? | Page 14- 19 |
| Clarity of major themes | 31 | Were major themes clearly presented in the findings? | Page 13-14 |
| Clarity of minor themes | 32 | Is there a description of diverse cases or discussion of minor themes? | Page 14- 19 |

Developed from: Tong A, Sainsbury P, Craig J. Consolidated criteria for reporting qualitative research (COREQ): a 32-item checklist for interviews and focus groups. International Journal for Quality in Health Care. 2007. Volume 19, Number 6: pp. 349 – 357
